# The longitudinal association between objectively measured physical activity and mental health among Norwegian adolescents

**DOI:** 10.1186/s12966-021-01211-x

**Published:** 2021-11-16

**Authors:** Ingeborg Barth Vedøy, Knut Ragnvald Skulberg, Sigmund Alfred Anderssen, Morten Wang Fagerland, Hege Eikeland Tjomsland, Miranda Thurston

**Affiliations:** 1 Inland Norway University of Applied Sciences, Postboks 400, 2418 Elverum, Norway; 2 The Norwegian school of Sport Sciences, Postboks 4014 Ullevål stadion, 0806 Oslo, Norway

**Keywords:** Physical activity, Accelerometry, Mental wellbeing, Mental health problems, Adolescence, Prospective cohort study

## Abstract

**Background:**

Mental health among young people in many countries, including Norway, seems to be deteriorating. Physical activity (PA) has been positively associated with mental health. However, methodological issues related to study design and measurement of PA and mental health outcomes currently limits our understanding of the relationship. The purpose of the present study is to explore the prospective relationship between objectively measured PA and mental health outcomes. More specifically, volume (total PA), intensity (moderate-to-vigorous PA [MVPA]) and sedentary behaviour (SED) were explored in relation to mental health problems (MHP) and mental wellbeing (MWB).

**Methods:**

Data from 599 adolescents (54.4% female, mean age at baseline ±SD 13.3 ± 0.3 years) were collected annually during their 3 years (T1, T2 and T3) at lower secondary school. PA was measured using accelerometry. MWB was measured using the ‘Warwick-Edinburgh Mental Wellbeing Scale’ and MHP by the ‘Strengths and Difficulties Questionnaire’. Multiple linear regression was performed to explore relationships between changes in PA/SED (between T1-T3) and MWB/MHP (at T3). The term ‘movement categories’ was used to refer to components on the movement continuum and includes volume (total PA), intensity (MVPA) and SED.

**Results:**

Among boys, any increase in SED was positively associated with MWB (β = 0.05, 95% CI: 0.01 to 0.10), whereas a small positive association between an increase in total PA (volume) and MWB was found among girls (β = 1.13, 95% CI: 0.05 to 2.21). There were no associations between changes in any movement categories [total PA (volume), MVPA, SED] and score on MHP at T3, neither for girls nor boys.

**Conclusion:**

This study provided no clear evidence of any association between change in volume or intensity of PA and MHP among an overall healthy adolescent study sample. There was, however, evidence of a relationship between increased SED and MWB among boys and increased volume of PA and MWB among girls. The relationship between movement categories and mental health may depend on the measurement used to assess both PA/SED and variables of mental health. Future research would be strengthened by researchers clarifying what construct of mental health is being used and measured.

**Supplementary Information:**

The online version contains supplementary material available at 10.1186/s12966-021-01211-x.

## Introduction

Mental health among young people has received increasing attention in recent years especially given its significance for a healthy transition to adulthood [1]. Yet international trends from high-income countries suggest their mental health is deteriorating, particularly among adolescent girls [2–4]. These trends are reflected in the increase in diagnosis and treatment of mental disorders over the past few decades [2]. In 2019, mental disorders represented the 4th leading global cause of disability-adjusted life-years among youth (10–24 years), contributing significantly to adolescents’ overall health burden [5]. There are few global estimates of the prevalence of mental health problems (MHP). In Norway, however, it is estimated that 15–20% of children and adolescents have reduced functions due to MHP [6]. Trends in Norway show that MHP vary according to age and sex, with older adolescent girls showing a larger increase in MHP over time than younger boys [7]. Although trends are complex, the apparent deterioration in young people’s mental health has stimulated extensive research into risk and protective factors. The role of physical activity in enhancing mental health and preventing MHP during adolescence has received particular attention as this is a period during which not only most mental disorders occur [8] but also levels of physical activity (PA) decline [9, 10]. Pooled data from the International children’s accelerometry database show that the PA level declines by 5.8% (4.7% in boys, 6.8% in girls) annually relative to the average PA level at 12 years [11].

There is evidence from systematic reviews as well as ‘reviews of reviews’ to indicate that PA is positively associated with various indicators of mental health among children and adolescents [12–15] and that relationships might be more consistent and robust for PA at higher intensities, (moderate-to-vigorous intensity PA [MVPA]) [16]. However, the strength of these relationships is weak to moderate. Methodological weaknesses in study design and measurement of PA have been put forward as limitations of the current knowledge base [13, 15]. Research using stronger study designs (longitudinal studies) and objective measures of PA have been called for to further clarify the relationship [14]. Alongside these methodological concerns, the conceptualization of mental health has increasingly been recognised as multidimensional, comprising both negative (e.g. depression and anxiety) and positive dimensions (e.g. mental wellbeing [MWB] and self-esteem) [14]. According to Westerhof and Keyes’ [17] two continua model, MHP or mental disorders and MWB are presented as two distinct but related dimensions. As they explain, the absence of negative indicators does not necessarily imply the presence of high levels of wellbeing and vice versa. The majority of research in the PA field to date has focused on negative indicators of mental health, such as MHP or mental disorders.

To the best of our knowledge, five longitudinal studies (including 1–3 year follow-up) have sought to address these methodological concerns among adolescents [18–22]. All these studies used accelerometry to measure PA. The relationship between PA and MHP was addressed in 4/5 studies, all of which found no association [18–21]. Within the PA-MWB relationship the findings were mixed showing both positive [22] or no [18] association. The heterogeneity of these studies should be noted, however. Not only have they been carried out on samples of young people from various countries where population levels of mental health and related social norms may be different, they have also used diverse instruments to measure MHP and MWB.

Recently, more attention has also been paid to the relationship between sedentary behaviours (SED) and indicators of mental health, and the role of SED in the mental health of young people was explored in a recent systematic review [15]. This found evidence of an inverse association between SED and satisfaction with life and happiness, as well as a positive association between SED and MHP. The authors underline the need for future research to explore relationships of SED and other indicators of MWB, for which there is currently minimal evidence.

The purpose of this study was therefore to explore the prospective relationship between objectively measured PA and mental health outcomes. More specifically, volume (total PA), intensity (MVPA) and SED were explored in relation to MHP and MWB. Further, the study sought to explore whether different PA transitions (see below) were associated with MHP or MWB.

## Methods

### Study design and participants

**Fig. 1:**
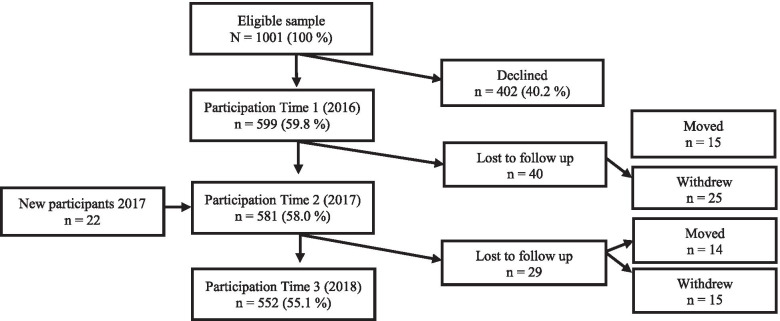
Flowchart of the study population and participation

In 2016, all students starting 8th grade (12–13 years) in 11 lower secondary schools were invited to participate in a prospective cohort study. The schools were recruited from two counties on the east and west side of Norway and were selected on the basis of school size, type of school (grades 1–10 or 8–10) location (urban, suburban and rural), socio-economic status and the school’s average score on National tests. Detailed description of the methodology has been published elsewhere [23]. Annual data collection took place during the first semester (i.e. Sept-Jan) of 8th, 9th and 10th grade. Figure 1 illustrates students’ participation.

### Measurements

#### Physical activity

Physical activity was measured objectively, using ActiGraph GT3X+/bt (LLC, Pensacola, Florida, USA). The monitor was placed on the right hip in an elastic belt. The participants were asked to wear it for seven consecutive days. To ensure correct placement, research staff attached the monitor during the first school visit. The participants were instructed to wear the monitor for all waking hours, except while doing water activities. The monitor was initialised to start recording at 06:00 the following day. After excluding data recorded between 00:00 and 06:00 and all intervals of ≥20 consecutive minutes of no recording (non-wear), accelerometer data were included if the participants had ≥480 min of valid activity recordings each day. A minimum of 4 days of valid data are recommended to achieve satisfactory reliability [24]. However, as analyses showed that participants with 2–3 days (Time 1 and 2 [T1 and T2]) and 1–3 days (Time 3 [T3]) of valid measurements were not significantly different from those with ≥4 days, these were included in further analyses. Data were downloaded in a 10 s epoch but aggregated up to a 60 s epoch for the intensity specific analysis. As an overall measure of PA volume (total PA), average counts·min^− 1^ (CPM) throughout the assessment period were used. We defined SED, light intensity PA (LPA) and MVPA as < 100 CPM, 100–1999 CPM and ≥ 2000 CPM respectively. Further, the Norwegian recommendation for PA ‘accumulation of an average of ≥60 min MVPA/day’ was used to categorise participants into adhering/not adhering to national recommendations [25]. The use of these accelerometer processing criteria were in accordance with previous Norwegian research conducted on similar age groups [10].

Because there may be differences between those keeping active or moving from inactive to active, and those remaining inactive or moving from active to inactive, analyses of PA transitions were conducted. PA transition profiles were created based on the participants adhering/not adhering to national PA recommendations in T1 and T3. ‘Active maintainers’ consist of those adhering to PA recommendations in T1 and T3, ‘move to inactive’ consist of those adhering to PA recommendations in T1, but not in T3, ‘move to active’ consist of those not adhering to PA recommendations in T1, but adhered to them in T3, and ‘inactive maintainers’ consist of those not adhering to PA recommendations in T1 nor in T3.

#### Mental health problems

MHP were measured using the Strengths and Difficulties Questionnaire (SDQ). The instrument consists of 25 questions divided into 5 scales of 5 questions each (Emotional problems scale, Conduct problems scale, Hyperactivity scale, Peer problems scale and Prosocial scale). Each item can be addressed “not true”, “somewhat true” or “certainly true”. All scales, except the Prosocial scale, are summed to create a ‘total difficulty score’ ranging from 0 to 40 where a higher score indicates poorer mental health [26]. The instrument has satisfactory reliability (Cronbach α = 0.8) and validity (OR 6.6 for a mental disorder for the 10% highest ‘total difficulties’ scores) among community samples of children and adolescents [27]. The current study used the established Norwegian translation. The continuous ‘total difficulty score’ was labelled ‘MHP’ and used as an outcome variable in all analyses.

#### Mental wellbeing

The Warwick-Edinburg Mental Wellbeing Scale (WEMWBS) was used to assess MWB. This instrument comprises 14 positively loaded items using a 5-point Likert scale (1 = ‘none of the time’ to 5 = ‘all of the time’). Responses were summed to create an overall wellbeing score ranging from 14 to 70, where a higher score indicates higher levels of wellbeing. The instrument is a psychometrically robust scale showing good content validity and high test-retest reliability [28]. The scale is validated for use among adolescents from 13 years of age and onwards [29] including Norwegian adolescents [30]. The current study used the established Norwegian translation with minor linguistic amendments. The continuous score was labelled ‘MWB’ and used as an outcome variable in all analyses.

#### Covariates

As reported in previous literature, the covariates that might confound the relationship of interest are: socio-economic status (SES) [31], body mass index (BMI) [32] and seasonality [33].

The Family affluence scale was used as a measure of SES. The scale measures material affluence and is a widely used proxy for SES [34]. A score of *relative family affluence* can be derived by summing scores on all answers and categorising them into three broader groups (the lowest 20%, the middle 60% and the highest 20%).

BMI was objectively measured and calculated using weight and height (kg·m^− 2^), measured to the nearest 0.1 kg (Seca 877, SECA GmbH, Hamburg, Germany) and 0.5 cm (wall-mounted measuring tape), respectively. Participants were asked to remove shoes and sweaters before measurement. Bodyweight measures were adjusted by subtracting 0.3 kg to account for clothing.

Seasonality was defined by two categories depending on the time of data collection at the different schools (1: Sept-Oct, 2: Nov-Jan).

### Ethics

The study was registered with the Norwegian Centre for Research Data (project no. 48192). Written informed assent from participants and consent from their legal guardians was obtained prior to data collection.

### Data analyses

Statistical analyses were performed using IBM SPSS Statistics for Windows, Version 24.0 and Stata Statistical Software, version 16.0 (Copyright 1985–2019 StataCorp LLC), Texas 77,845 USA. Descriptive data are presented as frequencies, mean and SD where appropriate (Tables 1 and 2). Hayes’ Process Macro v3.5 for SPSS was used to explore interactions by sex. As analysis showed an interaction, data were analysed separately for boys and girls. Paired T-tests were used to explore differences over time between variables at T1 and T3 (Table 1). To explore correlations between MHP and MWB, Pearson’s bivariate correlation analyses with 95% confidence intervals (CIs) based on the Fisher Z-transformation were used.

**Table 1:** Descriptive characteristics of the study sample by sex, all time points (mean ± SD unless otherwise specified)

Characteristic	Time 1 (2016) ***n*** = 599	Time 2 (2017) ***n*** = 581	Time 3 (2018) ***n*** = 552
**Age**			
Boys	13.4 (0.3)	14.4 (0.3)	15.4 (0.3)
Girls	13.3 (0.3)	14.3 (0.3)	15.3 (0.3)
**Height (cm)**			
Boys	163.9 (8.6)	171.3 (8.2)	176.9 (7.5)
Girls	161.7 (6.9)	165.0 (6.6)	166.3 (6.4)
**Weight (kg)**			
Boys	53.0 (11.8)	60.7 (13.5)	66.8 (13.6)
Girls	52.8 (9.9)	56.6 (9.8)	59.2 (9.8)
**BMI (kg/m**^**2**^**)**			
Boys	19.6 (3.3)	20.5 (3.7)	21.3 (3.7)**
Girls	20.0 (3.1)	20.8 (3.0)	21.4 (3.0)**
**MHP**			
Boys	9.7 (5.4)	10.1 (5.3)	10.4 (5.2)
Girls	10.0 (5.1)	11.3 (5.8)	12.0 (5.3)**
**MWB**			
Boys	57.1 (9.1)	57.3 (9.4)	55.9 (9.6)
Girls	55.0 (8.9)	52.2 (10.5)	50.6 (11.0)**
**Total PA (cpm/d)**			
Boys	474.4 (178.7)	476.8 (177.5)	460.9 (204.7)
Girls	402.2 (118.6)	399.9 (127.5)	383.5 (138.3)*
**SED (min/d)**			
Boys	548.4 (74.4)	541.7 (78.7)	542.1 (99.6)*
Girls	565.7 (55.4)	561.3 (64.6)	564.1 (69.6)
**LPA (min/d)**			
Boys	172.2 (34.1)	167.6 (34.3)	140.8 (37.3)**
Girls	161.4 (32.3)	153.9 (32.2)	137.6 (30.4)**
**MVPA (min/d)**			
Boys	63.9 (22.9)	62.4 (24.3)	58.1 (26.7)*
Girls	54.0 (18.5)	53.6 (19.4)	50.1 (21.4)*
**Meeting act.rec (%)**			
Boys	53.3	53.6	43.8
Girls	36.3	37.1	29.5

*Note. BMI* Body mass index, *MHP* Mental health problems, *MWB* Mental wellbeing, Total PA (cpm/d) = average daily counts per minute, SED = average daily sedentary behaviour, LPA = average daily light physical activity, MVPA = average daily moderate to vigorous physical activity, Meeting act.rec = meeting national recommendations for PA, min/d = minutes per day

* Significant change of variable between T1 to T3, *p* ≤ .05. Tests are performed on subsamples with valid measurements on outcome variable in T1 and T3 (*n* = 177–269)

** Significant change of variable between T1 to T3, *p* ≤ .001. Tests are performed on subsamples with valid measurements on outcome variable in T1 and T3 (*n* = 177–269)

**Table 2:** Change scores (SD) for movement categories [total PA (volume), MVPA, SED] between T1-T3

	T1 to T2^a^	T2 to T3^b^	T1 to T3^c^
MVPA^d^	0.2 (19.1)	−3.9 (21.7)	−4.0 (22.8)
SED^d^	−7.4 (36.9)	2.5 (39.6)	−4.4 (41.7)
Total PA (volume)^e^	5.8 (129.5)	−18.6 (147.4)	−14.5 (157.4)

^a^ *N* = 471 (Number of participants with valid data at time T1 and T2)

^b^ *N* = 426 (Number of participants with valid data at time T2 and T3)

^c^ *N* = 438 (Number of participants with valid data at time T1 and T3)

^d^ Variables are adjusted for mean wear time and presented as change in minutes

^e^ Variable is presented as change in mean counts per minute

To explore whether changes in movement categories [total PA (volume), MVPA and SED] from T1 to T3 were associated with MWB and MHP at T3, multiple linear regression analyses were used. Change scores (Δ) were created by subtracting T3-values from T1-values, creating new change variables that entered the model. The models were adjusted for T1-variables of MHP/MWB, BMI, SES, season of data collection. Further, all analyses were adjusted for school-level clustering and all analyses of ΔMVPA and ΔSED were adjusted for each other and wear time of the accelerometer (Table 3). As the associations can be bi-directional, the models were reversed, creating change scores of the mental health variables, and exploring their relationship with the different movement categories at T3 (Additional files 1 & 2).

**Table 3:** Association between change in movement categories and mental health outcomes at Time 3 among boys (*n* = 139–159) and girls (*n* = 196–222)

	Crude						Adjusted					
	MWB			MHP			MWB			MHP		
	β	95 %CI	p	β	95 %CI	p	β	95 %CI	P	β	95 %CI	P
**ΔTotal PA (volume)**^**a**^												
Boys	-.638	-1.48, .210	.139	.306	-.101, .713	.140	-.515	-1.09, .059	.074	.007	-.424, .439	.971
Girls	.880	-.133, .019	.088	-.129	-.620, .361	.606	1.13	.045, 2.21	**.043**	-.117	-.546, .312	.555
**ΔMVPA**^**b**^												
Boys	-.043	-.106, .019	.172	.025	-.005, .055	.102	.030	-.043, .104	.380	-.030	-.083, .023	.239
Girls	.065	-.002, .132	.057	-.018	-.050, .015	.290	.094	-.100, .287	.304	-.030	-.082, .021	.223
**ΔSED**^**c**^												
Boys	.048	.012, .084	**.010**	-.023	-.040, -.006	**.010**	.053	.009, .100	**.023**	-.022	-.055, .011	.169
Girls	-.014	-.048, .020	.422	.002	-.015, .019	.811	.010	-.096, .117	.832	-.010	-.031, .011	.310

*Note*: ΔTotal PA (volume) = change in total physical activity (volume), ΔSED = change in average daily sedentary behaviour, ΔMVPA = change in average daily moderate to vigorous physical activity, *MHP* = Mental health problems, *MWB* = Mental wellbeing

^a^ Adjusted for baseline MHP/MWB, baseline BMI, baseline socio-economic status, baseline season of data collection and cluster sampling. All measures are scaled up 100 times showing changes in dependent variables occurring after changes of 100 CPM.

^b^ Adjusted for accelerometer wear time, ΔSED, baseline MHP/MWB, baseline BMI, baseline socio-economic status, baseline season of data collection and cluster sampling

^c^ Adjusted for accelerometer wear time, ΔMVPA, baseline MHP/MWB, baseline BMI, baseline socio-economic status, baseline season of data collection and cluster sampling

To explore the influence of PA transitions, dummy variables of the different PA transition groups were created before entering the models alongside T1-variables of MHP/MWB, BMI, SES, season of data collection and sex. Associations between variables of PA transitions and score on MHP or MWB in T3 for the different groups were analysed using multiple linear regression (Tables 4 and 5). Further, post-hoc analyses of change in minutes of MVPA between T1 and T3 for the different PA transition profiles was conducted using paired T-test (Additional file 4).

**Table 4:** Inactive maintainers vs three transition profiles on mental health problems and mental wellbeing at Time 3 (linear regression results)

	Inactive maintainers (***n*** = 202)	Move to inactive (***n*** = 83)			Move to active (***n*** = 53)			Active maintainers (***n*** = 99)		
		β	95%CI	p	β	95%CI	p	β	95%CI	P
MHP^a^	ref.	1.11	−0.24, 2.45	.106	2.07	0.44, 3.69	**.013**	−0.09	−1.37, 1.19	.887
MHP^b^	ref.	1.30	0.11, 2.48	.**033**	0.47	−1.00, 1.93	.533	0.41	−0.73, 1.54	.480
MWB^a^	ref.	−0.45	−3.24, 2.35	.755	1.03	−2.41, 4.46	.557	1.62	−1.01, 4.25	.227
MWB^b^	ref.	−1.59	−4.26, 1.07	.240	1.24	−2.05, 4.52	.460	0.77	−1.72, 3.26	.543

*Note.* MHP^a^ ref. = 10.50, MHP^b^ ref. = 0.74. MWB^a^ ref. = 52.32, MWB^b^ ref. = 36.38. MHP = Mental health problems (T3), MWB = Mental wellbeing (T3)

^a^ Crude

^b^ Adjusted for baseline MHP/MWB, baseline sex, baseline BMI, baseline socio-economic status and baseline season of data collection

**Table 5:** Active maintainers vs two transition profiles on mental health problems and mental wellbeing at Time 3 (linear regression results)

	Active maintainers (***n*** = 99)	Move to inactive (***n*** = 83)			Move to active (***n*** = 53)		
		β	95%CI	***p***	β	95%CI	***p***
MHP^a^	ref.	1.20	−0.34, 2.74	.127	2.16	0.37, 3.95	**.018**
MHP^b^	ref.	0.89	−0.46, 2.23	.195	0.06	−1.53, 1.65	.944
MWB^a^	ref.	−2.07	−5.24, 1.11	.201	−0.59	−4.34, 3.15	.756
MWB^b^	ref.	−2.36	−5.31, 0.58	.116	0.47	−3.06, 3.98	.795

*Note.* MHP^a^ ref. = 10.40, MHP^b^ ref. = 1.15. MWB^a^ ref. = 53.94, MWB^b^ ref. = 37.15. MHP = Mental health problems (T3), MWB = Mental wellbeing (T3)

^a^ Crude

^b^ Adjusted for baseline MHP/MWB, baseline sex, baseline BMI, baseline socio-economic status and baseline season of data collection

In this study WEMWBS was assessed in a six-point Likert scale from *not at all* (0) to *all the time* [5]. To be able to compare results with international data, the following equation was applied: ‘WEMWBS 5-point score = 1 + ((4/5)*WEMWBS 6-point score)’. As the SDQ guidelines enables imputations, these have been performed for the instrument according to standard practice. While analysis showed variables not to be missing completely by random for WEMWBS, imputations were not performed for this instrument.

## Results

### Descriptive characteristics

Descriptive statistics on key variables over the 3 years of study are shown in Table 1. Significant differences between T1 and T3 were seen for all variables among girls, except SED. For boys, significant differences were evident for BMI, SED, LPA and MVPA. The proportion of students adhering to national recommendations for PA spanned from 29.5 to 36.3% from T1 to T3 among girls, and from 43.8 to 53.3% among boys in the same period. Compared to nationally representative data among Norwegian 15-year olds from 2018 (girls: 44.1%, boys: 55.3%) [35], the current study population was less active than the national average. The proportion of students with MHP-scores categorised as normal remained high (boys: 85–80%, girls: 84–76%) throughout the three time points (see Additional file 4). The same pattern was evident for MWB, where the mean scores remained high (boys: 57.1–55.9, girls: 55.0–50.6), the score distributions being positively skewed throughout the three time points (see Additional file 5). The correlations between MHP and MWB were moderate throughout the study (T1: r = − 0.44, 95% CI -0.51, − 0.37; T2: r = − 0.45, 95% CI -0.52, − 0.38; T3: r = − 0.53, 95% CI -0.60, − 0.47) indicating that they are distinct, but related dimensions of mental health.

Change scores for movement categories for the three timepoints (Table 2) show the same pattern, where the change between T1 to T3 is similar to that of T2-T3 and thus adequately reflects the overall changes over time in this study. Further, the variation in the standard deviations (SD) in Table 2 far exceeds any changes over time. This is exemplified by the values and SDs of total PA (volume); 5.8 (129.5), − 18.6 (147.4) and − 14.5 (157.4).

### Prospective analyses between movement categories and MHP/MWB

Prospective analyses showed that an increase in SED was positively associated with MWB score at T3 among boys (Table 3). The magnitude of the association translates to a daily increase in SED of 60 min from T1 to T3 being equivalent to a 3.2 points higher score in MWB. Among girls, an increase in total PA (volume) was positively associated with MWB score at T3 (Table 3). As a change of 1 cpm is too small to reflect any meaningful change in outcome variable, the value is scaled up 100 times, showing changes in outcome variable occurring after changes of 100 cpm. This change corresponds to an increase in daily activity level of approximately 20 %. Such an increase in activity level will generate an increased score in MWB of 1.13 points. In the adjusted models, no associations were found between changes in any movement categories [total PA (volume), MVPA, SED] and score on MHP at T3, neither for girls nor boys (Table 3). In the reversed model (Additional files 1 & 2), changes in MHP/MWB were used as predictors and measures of movement categories [total PA (volume), MVPA and SED] at T3 were the outcomes. Neither of these analyses showed any significant relationships between the variables of interest among boys or girls, posing no evidence of a bi-directional association.

### PA transition analyses

Analyses of differences in MHP and MWB at T3 for different PA transition profiles showed a significant difference in MHP-score at T3 between ‘inactive maintainers’ and ‘move to active’ (*p* = .013), and between ‘active maintainers’ and ‘move to active’ (*p* = .018) in the crude analyses (Tables 4 and 5). However, both differences disappeared in the adjusted analyses. In the adjusted analyses, a significant difference in MHP-score at T3 was found between ‘inactive maintainers’ and ‘move to inactive’, where ‘move to inactive’ scored 1.3 units higher than ‘inactive maintainers’ (*p* = .033).

## Discussion

The purpose of this study was to explore the longitudinal relationship between objectively measured PA and mental health outcomes. Our main results showed that an increase in SED from T1 to T3 was associated with a higher MWB score at T3 for boys. Among girls, results indicated that an increase in total PA (volume) from T1 to T3 was associated with a higher score on MWB in T3. Reverse analyses, where the relationship between change of MHP/MWB and movement categories [total PA (volume), MVPA and SED] at T3 were explored, showed no indication of any bi-directional relationship. Descriptive analyses (Table 2) demonstrated that changes from T1 to T3 adequately reflected the overall changes over time. As the study was exploratory and not confirmatory, change scores were used to address the aims of the study adequately.

The results with respect to the positive association between SED and MWB for boys, contradicts results from a previous systematic review [15]. However, this review includes studies which have measured MWB using different instruments as well as setting no criteria for objective measures of movement patterns. Results might thus not be directly comparable with the current study. To the best of our knowledge, this is the first study to explore the relationship between objectively measured SED and MWB (measured by WEMWBS) among adolescents. Although there is yet no consensus regarding what constitutes an important change in WEMWBS, the instrument has been found to be responsive at both individual and group/population level [36]. At an individual level, a change of 3 or more units (1 SEM) has been interpreted as important [36]. The current study found a 60 min increase in SED to be equivalent to 3.2 points increase in WEMWBS. We interpret this as an important change in MWB among boys in this study. As SED was assessed using accelerometry, we do not know the context of this behaviour. Previous research has shown that distinct sedentary behaviours might associate differently with variables of mental health, e.g. screen-based vs non-screen-based sedentary behaviour [37]. Moreover, recent research has found differences between passive screen time, which has been associated with mood and anxiety disorders, and active screen time which has not [38]. The social interplay which gaming, for example offers, might serve as one explanation for the positive association between SED and MWB in the current study. How SED relates to MWB remains an important focus for future research.

Among girls, results showed that an increase in total PA (volume) was associated with a higher score on MWB. As a rather large increase in PA-levels (about 20%) was equivalent to a somewhat small increase in MWB score (1.13 points), it was not considered to constitute an important change based on the suggested criteria [36]. Although weak, this association contradicts the results of Bell et al. [18] who found no evidence of an association between total PA (volume) and score on MWB (measured using the same instrument) among the same age group. However, Bell et al. [18] measured PA at baseline only and explored changes in outcome 3 years later. The results cannot, therefore, associate changes in PA with MWB score. The relationship between total PA (volume) and MWB among girls in particular, is thus in need of further exploration.

There were no significant associations between movement categories and MHP among girls or boys in the current study. These results are in line with previous research which found no association between volume- [18–20] or intensity [18, 20, 21] of objectively measured PA and measures of MHP among adolescents. This might suggest that MHP are better explained by variables other than change of movement categories. Toseeb et al. [20] discuss whether associations between PA and MHP might be small or non-existent during the adolescent years, as research more frequently reports positive associations in adult populations. Throughout all time points of this study, the sample means were low for MHP and high for MWB. Stronger effects of PA on mental disorders have been found in clinical samples [14], which might indicate that the overall good level of mental health in the study sample makes any contribution of PA negligible. Opdal et al. [21] also argue that in a study sample where few adolescents experience change in their mental health, it is difficult to find associations with change in PA levels. Although the MHP score increased significantly among girls between T1 and T3, the mean scores of boys and girls in the current study were all within the normal range, and thus indicative of a mentally healthy study sample. The same pattern was also present among British adolescents of the same age, where the mean scores were within the normal range and no association was found between volume or intensity of PA and MHP [18].

Analyses exploring the longitudinal associations between PA and MHP through categorisation of participants into different PA transition profiles nevertheless found participants belonging to the ‘move to inactive’ profile to have a significantly higher score on MHP than ‘inactive maintainers’. Previous research has shown that participants changing from an active to a sedentary profile showed a larger decrease in positive affect when compared with profiles that showed no reduction in PA [22]. There were, however, no equivalent differences between ‘active maintainers’ and ‘move to inactive’ in the current study, providing no clear evidence for such an explanation. In addition, the 95% CI for the β ‘move to inactive’ included a very small effect (0.11), which indicates that the ‘move to inactive’ group is not that different from the ‘inactive maintainers’. There were no differences in MWB between different PA transition profiles, further elucidating the weak relationship between PA and MWB in the current study sample. These results contradict those of Sánchez-Oliva et al. [22] who found that belonging to active profiles (participants engaging in lower levels of SED and higher levels of PA) was associated with better MWB (measured by HRQoL and positive affect) compared to belonging to sedentary profiles (participants engaging in higher levels of SED and lower levels of PA). However, we used adhering/not adhering to national PA recommendations at T1 and T3 as the criteria for placement within the different PA profiles. As small changes in activity level may tip a participant from one group to another (e.g. 59 min of MVPA in T1 and 61 min of MVPA in T3), there might not have been a large enough change to produce effects on variables of MHP and MWB. Post-hoc analysis was therefore conducted to explore whether the average MVPA for the different PA transition profiles varied significantly between T1 and T3 (see Additional file 3). This analysis showed large and significant changes in MVPA within all groups except Active maintainers. Small changes between T1 and T3 can thus not explain the weak relationship with mental health outcomes.

### Methodological considerations

There are several strengths to this study. First, the longitudinal design with very high adherence and the use of accelerometry to measure PA alongside validated instruments to measure variables of mental health are key strengths. Both predictor variables and outcomes were measured at all time points. This created a strong longitudinal line of data and enabled exploration of the temporal nature of potential relationships, as well as taking into account the changing patterns of PA, which was called for in a recent study [18]. Furthermore, we controlled for important covariates. However, both PA and mental health are multifactorial and complex constructs, which are influenced by several known and unknown factors [12, 39]. Consequently, there are likely to be additional factors, that we have no measure of, which may override the possible associations between PA and variables of mental health. As there were few significant findings this may suggest that mental health outcomes are better explained by other factors than change in movement patterns, or that our measurements were not sufficiently sensitive to detect patterns that might be present. Although providing an objective measure of PA, accelerometry also has limitations. Monitors are not completely waterproof and must be removed while doing water activities. Further, they underestimate the energy cost for cycling, activities on a gradient and arm-intensive activities [40]. Registration of time spent swimming and cycling was conducted to account for some of these issues. As the main proportion of pupils reported 0–1 h of both activities, swimming and cycling were not considered to contribute to underestimating the true activity level, at least not to a large degree. In addition, accelerometers might not be sufficiently sensitive to capture patterns of SED because of their limitations in the measurement of posture [37], which may have led to a less precise measure of SED among the study sample. Moreover, despite being validated instruments, the measures of mental health may also introduce limitations. The potential insensitivity to change over time (SDQ), as well as problems with discriminating sufficiently within high (SDQ and WEMWBS) and low scores (SDQ) [30, 41], may limit their ability to assess associations within overall healthy study samples. Further, WEMWBS score was obtained using a 6-point scale instead of the validated 5-point scale. Although the scores have been corrected using a customized equation, the presentation of a 6-point scale instead of the validated 5-point scale may have influenced the responses of the participants to some degree.

Although the non-random sampling prevents us from drawing definitive conclusions regarding generalisability, the sampling procedure nevertheless ensures that the participating schools are likely to be broadly representative of secondary schools found in Norway. However, because the participation rate was 60% at T1, and no drop-out analysis was conducted, this might have introduced some selection bias in our sample and hence findings. Nevertheless, the loss to follow-up was small, providing a longitudinal participation rate of 97.0 and 92.2% in T2 and T3 respectively when T1 is set as the eligible sample. Finally, analyses explored change scores between T1 to T3 allowing all changes of equal duration to be seen as similar regardless of baseline level. If changes of equal duration are not similar, this may have implications for the results.

## Conclusion

This study provided no clear evidence for any association between change of volume or intensity of PA and MHP among an overall healthy adolescent study sample. When MWB was the outcome, a weak association was found with change in total PA (volume), solely among girls, and an association with change in SED among boys. However, as objective measures alone cannot provide information about the context of the SED, future research should combine objective measures with subjective measures to enable such contextual information to be captured and potentially generate a more detailed picture of the relationship between SED and variables of mental health. Further, there is a need to increase the sensitivity of measures of mental health to improve their ability to discriminate within high and low scores, advancing our knowledge on the relationship between PA and variables of mental health in overall mentally healthy study samples. Lastly, as analyses show that MHP and MWB are distinct, but related dimensions of mental health, there is a need for researchers to be clear on what construct of mental health they are exploring, and how they operationalise their measurements.

## Supplementary Information

**Additional file 1: Figure 1.** Association between change in variables of mental health (2016-2018) and intensities of PA in 2018 among boys. (*N*= 139-159).

**Additional file 2: Figure 2.** Association between change in variables of mental health (2016-2018) and intensities of PA in 2018 among girls. (*N*= 196-222).

**Additional file 3: Table 1.** Differences in minutes in MVPA between T1 and T3 analysed by paired T-test.

**Additional file 4: Table 2.** Score distribution of SDQ within a 4-band categorization in T1-T3 (n + %).

**Additional file 5: Figure 3.** Score distribution of WEMWBS in T1-T3*.*

## Data Availability

The datasets generated and/or analysed during the current study are available from the corresponding author on reasonable request.

## Abbreviations

MHP: Mental health problems
MWB: Mental wellbeing
PA: Physical activity
LPA: Light intensity PA
MVPA: Moderate-to-vigorous intensity PA
SED: Sedentary behaviours
CPM: Average counts·min^− 1^
T1: Time 1
T2: Time 2
T3: Time 3
WEMWBS: The Warwick-Edinburg Mental Wellbeing Scale
SDQ: Strengths and Difficulties Questionnaire
BMI: Body mass index
CI: Confidence intervals
SES: Socio-economic status
