# Supplementary material for: The longitudinal association between objectively measured physical activity and mental health among Norwegian adolescents
Source: Int J Behav Nutr Phys Act. 2021 Nov 16;18:149. doi: 10.1186/s12966-021-01211-x (PMC8594230; doi:10.1186/s12966-021-01211-x)
Supplement: Supplementary file 1 — Additional file 1: Figure 1. Association between change in variables of mental health (2016-2018) and intensities of PA in 2018 among boys. (N= 139-159). [file 12966_2021_1211_MOESM1_ESM.docx]

β = -.610 (-5.81 to 4.59)

PA (Total PA, volume) 2018^a^

PA (MVPA) 2018^b^

ΔMHP score

ΔMWB score

SED 2018^b^

β = -.161 (-.841 to .521)

β = .091 (-1.07 to 1.26)

β = -1.54 (-4.74 to 1.65)

β = -.193 (-.660 to .274)

β = .533 (-.399 to 1.47)

Note: MHP = Mental health problems, MWB = Mental Wellbeing, MVPA = Moderate to Vigorous Physical activity, SED = Sedentary time ^a^ Adjusted for baseline sex, BMI, SES, season of data collection and cluster sampling
^b^ Adjusted for accelerometer wear time, baseline sex, BMI, SES, season of data collection and cluster sampling

**Additional file 1, figure 1.** Association between change in variables of mental health (2016-2018) and intensities of PA in 2018 among boys. (N= 139-159)
