# Supplementary material for: The longitudinal association between objectively measured physical activity and mental health among Norwegian adolescents
Source: Int J Behav Nutr Phys Act. 2021 Nov 16;18:149. doi: 10.1186/s12966-021-01211-x (PMC8594230; doi:10.1186/s12966-021-01211-x)
Supplement: Supplementary file 3 — Additional file 3: Table 1. Differences in minutes in MVPA between T1 and T3 analysed by paired T-test. [file 12966_2021_1211_MOESM3_ESM.docx]

**Additional file 3, table 1.** Differences in minutes in MVPA between T1 and T3 analysed by paired T-test.

|  | **n** | **T1 Mean MVPA (SD)** | **T3 Mean MVPA (SD)** | **t** | **p** |  |  |
| --- | --- | --- | --- | --- | --- | --- | --- |
| Active maintainer | 99 | 79.2 (15.1) | 79.7 (17.7) | 0.3 | .755 |  |  |
| Move to inactive | 83 | 74.7 (13.3) | 43.8 (11.3) | -15.0 | <.001 |  |  |
| Move to active | 53 | 46.2 (10.9) | 75.4 (17.3) | 9.3 | <.001 |  |  |
| Inactive maintainers | 202 | 42.8 (10.3) | 37.3 (11.7) | -6.0 | <.001 |  |  |
