# Supplementary material for: The longitudinal association between objectively measured physical activity and mental health among Norwegian adolescents
Source: Int J Behav Nutr Phys Act. 2021 Nov 16;18:149. doi: 10.1186/s12966-021-01211-x (PMC8594230; doi:10.1186/s12966-021-01211-x)
Supplement: Supplementary file 4 — Additional file 4: Table 2. Score distribution of SDQ within a 4-band categorization in T1-T3 (n + %). [file 12966_2021_1211_MOESM4_ESM.docx]

**Additional file 4, table 2**. Score distribution of SDQ within a 4-band categorization in T1-T3 (n + %).

|  | **T1 (2016)** | **T2 (2017)** | **T3 (2018)** |
| --- | --- | --- | --- |
| **‘Close to average’** (0-14) | 483 (84.0) | 404 (75.0) | 378 (77.5) |
| **‘Slightly raised’** (15-17) | 48 (8.3) | 66 (12.2) | 65 (13.3) |
| **‘High’** (18-19) | 14 (2.4) | 27 (5.0) | 32 (6.6) |
| **‘Very high’** (20-40) | 30 (5.2) | 42 (7.8) | 13 (2.7) |
| Total | 575 (100) | 539 (100) | 488 (100) |
