# Supplementary material for: The longitudinal association between objectively measured physical activity and mental health among Norwegian adolescents
Source: Int J Behav Nutr Phys Act. 2021 Nov 16;18:149. doi: 10.1186/s12966-021-01211-x (PMC8594230; doi:10.1186/s12966-021-01211-x)
Supplement: Supplementary file 5 — Additional file 5: Figure 3. Score distribution of WEMWBS in T1-T3. [file 12966_2021_1211_MOESM5_ESM.docx]

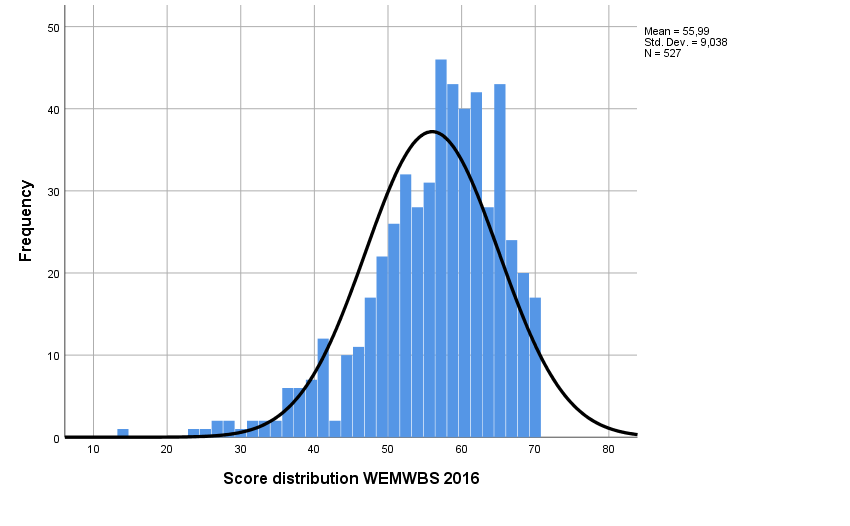

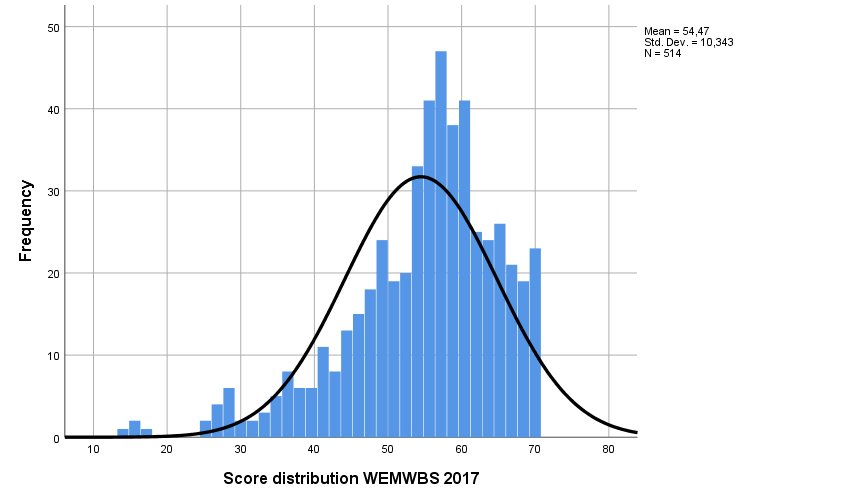

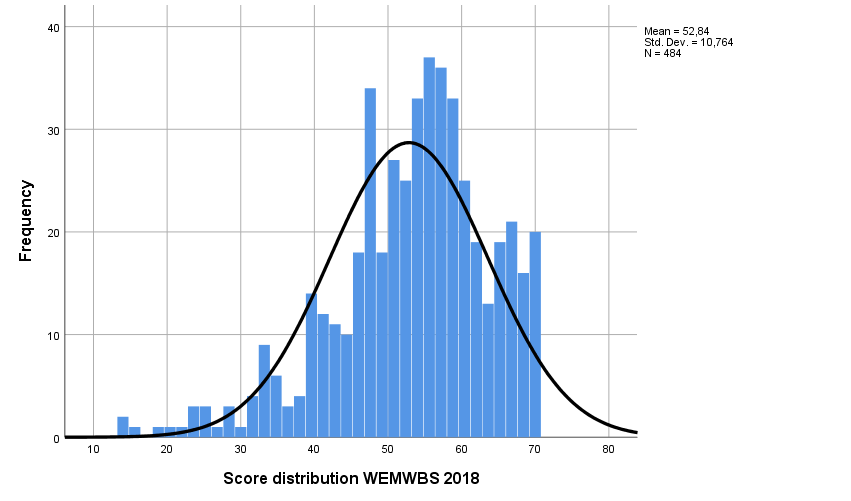


Note. Total WEMWBS score range = 14 - 70 points

**Additional file 5, figure 3**. Score distribution of WEMWBS in T1-T3.
